# Decline in Mast Cell Density During Diffuse Alveolar Damage in Idiopathic Pulmonary Fibrosis

**DOI:** 10.1007/s10753-021-01582-0

**Published:** 2021-10-22

**Authors:** Johanna Salonen, Mervi Kreus, Siri Lehtonen, Hannu Vähänikkilä, Minna Purokivi, Riitta Kaarteenaho

**Affiliations:** 1 Respiratory Medicine, Research Unit of Internal Medicine, University of Oulu, P.O. Box 8000, 90014 Oulu, Finland; 2 Medical Research Center (MRC) Oulu, Oulu University Hospital, P.O. Box 20, 90029 OYS Oulu, Finland; 3 Department of Obstetrics and Gynecology, PEDEGO Research Unit, Oulu University Hospital, University Hospital of Oulu, P.O. Box 23, 90029 OYS Oulu, Finland; 4 Infrastructure for Population Studies, Faculty of Medicine, Northern Finland Birth Cohorts, University of Oulu, Arctic Biobank, P.O. Box 8000, 90014 Oulu, Finland; 5 The Center of Medicine and Clinical Research, Division of Respiratory Medicine, Kuopio University Hospital, P.O. Box 100, 70029 KYS Kuopio, Finland

**Keywords:** mast cells, idiopathic pulmonary fibrosis, fibrosis, acute exacerbation, alveolar parenchyma.

## Abstract

Mast cells (MCs) are known to be involved in the pathogenesis of idiopathic pulmonary fibrosis (IPF), although their role in acute exacerbations of IPF has not been investigated. The aims of the study were to evaluate the numbers of MCs in fibrotic and non-fibrotic areas of lung tissue specimens of idiopathic pulmonary fibrosis (IPF) patients with or without an acute exacerbation of IPF, and to correlate the MC density with clinical parameters. MCs of IPF patients were quantified from surgical lung biopsy (SLB) specimens (*n* = 47) and lung tissue specimens taken at autopsy (*n* = 7). MC density was higher in the fibrotic areas of lung tissue compared with spared alveolar areas or in controls. Female gender, low diffusion capacity for carbon monoxide, diffuse alveolar damage, and smoking were associated with a low MC density. MC densities of fibrotic areas had declined significantly in five subjects in whom both SLB in the stable phase and autopsy after an acute exacerbation of IPF had been performed. There were no correlations of MC densities with survival time or future acute exacerbations. The MC density in fibrotic areas was associated with several clinical parameters. An acute exacerbation of IPF was associated with a significant decline in MC counts. Further investigations will be needed to clarify the role of these cells in IPF and in the pathogenesis of acute exacerbation as this may help to identify some potential targets for medical treatment for this serious disease.

**Supplementary Information:**

The online version contains supplementary material available at 10.1007/s10753-021-01582-0.

## INTRODUCTION

Idiopathic pulmonary fibrosis (IPF) is a severe interstitial lung disease of unknown cause, in which the phenomenon of acute exacerbation (AE) is responsible for significant mortality [1, 2]. The role of inflammation in the pathogenesis of IPF has been thought to be of minor importance, because anti-inflammatory drugs have not been demonstrated to be beneficial in the treatment of the disease [2, 3].

Mast cells (MCs) are immune cells whose significance in pulmonary diseases has been best characterized in allergic asthma; the role of MCs in other respiratory diseases is less clear [4, 5]. Peripheral MCs seem to exert immune-modulatory, pro-inflammatory, and pro-fibrotic effects in airways and lung parenchyma [5]. Table 1 given in Online Resource 1 presents the previous studies on MCs and their role in pulmonary fibroses. In vitro, MCs have been observed to induce both the proliferation and the activation of human fibroblasts [6]. On the other hand, it has been described in another study that fibroblasts in turn promoted MC activation and proliferation [7]. In a human lung fibroblast cell culture model, involving fibroblasts collected from IPF patients, both MCs and tryptase increased the release of mediators by fibroblasts which subsequently influenced epithelial migration [8].

A higher number of MCs have been observed in IPF compared with other interstitial lung diseases or normal controls [7, 9–15]. MCs of lung tissue have been shown to contain granules with fibrogenic mediators, such as basic fibroblast growth factor, transforming growth factor β1 (TGF-β1), histamine H1, tryptase and renin [7, 11, 13, 15–17]. Furthermore, the degree of fibrosis and the number of fibroblast foci have been observed to be positively correlated with the MC density in fibrotic lung tissue [6, 10, 11, 13, 16].

Although MCs appear to be involved in the pathogenesis of pulmonary fibrosis, the role of these cells in the development of AE-IPF has not been investigated. There were two aims of this study: (1) to evaluate the numbers of MCs in fibrotic and non-fibrotic areas of lung tissue specimens of IPF patients with and without an AE-IPF, and (2) to correlate the MC density with clinical parameters such as age, gender, pulmonary function test results, and smoking status. We also wanted to investigate whether the MC count was associated with the risk for mortality or the occurrence of AE-IPF.

## MATERIALS AND METHODS

### Patient Collection

The study cohort consists of 47 IPF patients treated in Oulu University Hospital or Oulaskangas Hospital in Northern Finland between 1991 and 2019. All study subjects had undergone a surgical lung biopsy procedure for diagnostic purposes. The lung tissue specimens taken at autopsy were also studied from seven subjects out of 47. Non-diseased lung tissue material from 12 non-smokers who had undergone lung cancer surgery served as controls (Table 2 in Online Resource 1). IPF had been diagnosed according to the international guidelines [2, 18]. The patients with a history of AE-IPF (*n* = 21) were identified either by applying the current criteria for AE-IPF in those subjects in whom lung tissue material had not been obtained during the episode of AE-IPF (*n* = 13), or on the basis of the surgical lung biopsy material, i.e., diffuse alveolar damage (DAD) in parallel with usual interstitial pneumonia (UIP) indicative of AE-IPF (*n* = 8) [1].

The surgical lung biopsy date, age at biopsy, pulmonary function test (PFT) results examined at a time near the biopsy date, antifibrotic drug use, medical treatment preceding AE-IPF in patients with DAD in a biopsy or autopsy specimen, and smoking data were collected systematically from the medical records. Patients with less than 5 pack-years of smoking history were regarded as non-smokers. Age and follow-up time were calculated by using the biopsy procedure date and death, transplantation or last follow-up date (11/8/2020). Death dates were collected from death certificates housed in the national registry of Statistics Finland.

### Mast Cell Staining and Quantification

Formalin-fixed and paraffin-embedded lung specimens were cut into 3.5 μm sections, de-paraffinized in xylene and rehydrated in a descending ethanol series. The staining was performed by using Dako REAL EnVision Detection System (Dako, Glostrup, Denmark). Microwave-stimulated antigen retrieval was performed in Tris–EDTA, pH9, and endogenous peroxidase was neutralized with peroxidase blocking solution (Dako). After the specimens had been incubated with a primary monoclonal mouse anti-human mast cell tryptase antibody (Clone AA1, M7052, Dako) diluted 1:200 in antibody diluent (Dako) for 30 min at room temperature, a biotinylated secondary HRP rabbit/mouse antibody (Dako) was added. The color was developed with 3,3′-diaminobenzidine (DAB, Dako) and counterstaining was performed with hematoxylin. As a negative control, the primary antibody was replaced by mouse isotype control (Invitrogen, Carlsbad, USA). The stained sections were digitized with Leica-Aperio AT2 (Leica Biosystems, Nussloch, Germany).

Digitized lung tissue specimens were examined by using virtual microscopy software (Aperio Image Scope, Version 12.4.3.5008, Leica Biosystems). The MCs were visualized maximally at 40 × magnification and were enumerated from ten randomly selected 0.16-mm^2^ areas in fibrotic areas of lung tissue in IPF and in normal alveolar areas of control subjects. Due to the small amount of fibrotic areas in surgical lung biopsy samples of two subjects, five, and eight 0.16-mm^2^ areas were assessed instead of ten. In addition, three 0.16 square millimeter areas of non-fibrotic alveoli were assessed from specimens of 33 IPF patients, in whom there was enough preserved lung tissue to permit this analysis, i.e., in study subjects with IPF, the numbers of mast cells in both fibrotic and non-fibrotic areas were counted. The MC density of fibrotic areas was the value used when the correlations with clinical parameters were calculated. J.S. calculated the MCs from all lung tissue specimens. Two other investigators (S.L. and M.K.), calculated the mast cell densities of 20 different study subjects. In addition, MC densities of honeycombing areas, fibroblast foci and areas of dense fibrosis were calculated from 12 representative samples (S.L. and M.K.).

### Statistical Analysis

IBM SPSS Statistics for Windows, Version 27.0 (Armonk, NY: IBM Corp.) was used to perform statistical analysis, and Origin(Pro), Version 2019b (OriginLab Corporation, Northampton, MA, USA), was utilized for preparing the graphs. Means and standard deviations were calculated for parameters that were normally distributed. Medians and interquartile ranges were determined for parameters that were not normally distributed. We used independent samples *t*-test or paired samples *t-*test to compare means when appropriate. The intraclass correlation coefficient was determined to evaluate the interrater reliability of MC counts. Survival analysis was performed by using Kaplan–Meier curves, and risk for earlier death or earlier episode of AE-IPF was evaluated by using Cox regression model. Medians and quartiles of MC densities were utilized to determine the cutoff values for Kaplan–Meier and Cox regression analyses. We included MC densities of SLB samples, not autopsy samples, in the Kaplan–Meier and Cox regression analyses.

## RESULTS

### Study Subjects

The characteristics of the study subjects are shown in Table 1. We examined surgical lung biopsies from 47 study subjects with IPF, of whom 44 subjects had a histology of UIP and three cases had UIP with DAD. We had additional autopsy lung tissue specimens from 7 out of 47 study subjects. Most patients (72%) were male and more than half were ex- or current smokers. Twenty-one patients out of 47 experienced an episode of AE-IPF during the follow-up time. Histological confirmation of AE-IPF was available from 8 patients, who had UIP with DAD either in surgical lung biopsy (*N* = 1), autopsy (*N* = 5) or both in autopsy and lung biopsy specimens (*N* = 2).

**Table 1:** Characteristics of the study subjects with IPF

Characteristic	*N* = 47
Age, years^a^	62 ± 7.7
Male	34 (72)
Surgical lung biopsy	47 (100)
UIP	44 (91)
UIP with DAD	3 (6.4)
Autopsy lung tissue specimen	7 (15)
UIP with DAD	7 (15)
Two separate lung tissue specimens (biopsy and autopsy)	7 (15)
UIP in biopsy and UIP with DAD in autopsy	5 (11)
UIP with DAD in both specimens	2 (4.3)
Smoking status at biopsy^b^	
Never-smoker	16 (34)
Ex-smoker	20 (43)
Current smoker	6 (13)
Pack-years of ever-smokers^c^	26 ± 11
FVC%^d^	74 ± 16
FEV1%^e^	79 ± 17
FEV1/FVC^d^	86 ± 6.0
DLCO^f^	55 ± 13
Antifibrotic drug therapy during follow-up	17 (36)
Pirfenidone	14 (30)
Nintedanib	3 (6.4)
Episode of AE-IPF during follow-up	21 (45)
Drug therapy preceding biopsy or autopsy in patients with DAD	
Antifibrotic drug	0
Corticosteroids	6 (13)
Some other anti-inflammatory drug^g^	0
Follow-up time (years)	3.9 (1.6 − 7.5)
Deceased or transplanted	36 (77)
Transplanted	4 (8.5)

Data is presented as mean ± standard deviation, median (interquartile range) and number of patients (%) when appropriate

*AE-IPF* acute exacerbation of idiopathic pulmonary fibrosis, *DAD* diffuse alveolar damage, *DLCO* diffuse capacity for carbon monoxide, *FEV1* forced expiratory volume at 1 s, *FVC* forced vital capacity, *UIP* usual interstitial pneumonia

^a^Age at biopsy date

^b^Smoking data of 5 patients was missing

^c^Pack-year data of 13 smokers or ex-smokers was missing

^d^Data of 9 patients was missing

^e^Data of 8 patients was missing

^f^Data of 10 patients was missing

^g^One patient had received azathioprine preceding hospitalization due to AE-IPF, but azathioprine was discontinued at the time of hospitalization

### Mast Cell Profile Differences Were Associated with Several Clinical Parameters

**Fig. 1:**
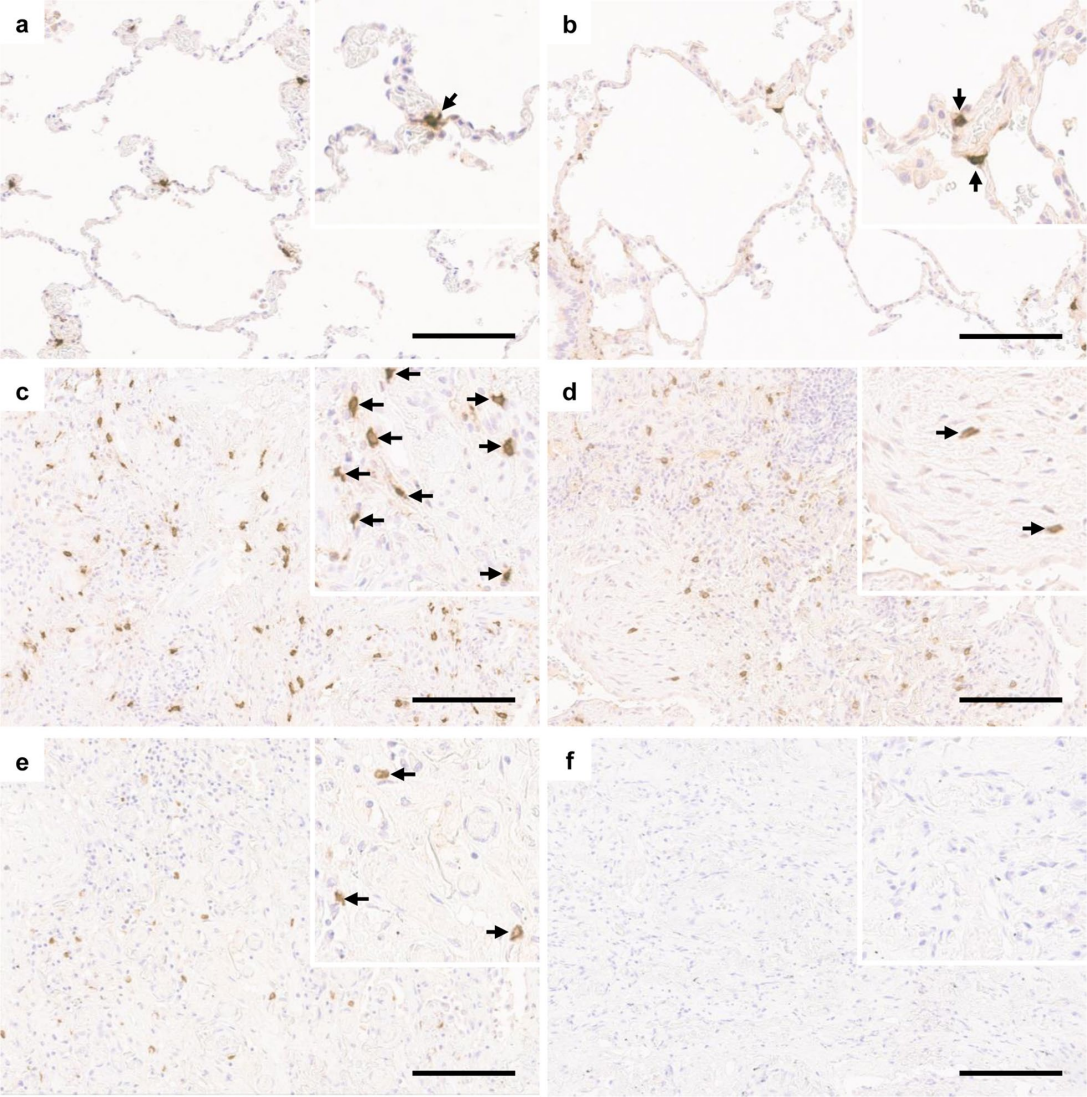
Immunohistochemical analysis of mast cells in idiopathic pulmonary fibrosis (IPF). Lung tissue sections were stained with mast cell tryptase antibody and positive cells are shown in brown color (arrows). **a** Normal control lung. **b** Spared alveolar tissue of IPF patient. **c** Fibrotic pulmonary tissue in the stable phase of the disease (surgical lung biopsy). **d** Fibroblast focus in the stable phase of the disease. **e** Fibrotic pulmonary tissue during an acute exacerbation (autopsy specimen). **f** Mouse isotype control. Scale bar 150 µm.

The MC densities in different types of lung tissue specimens are shown in Table 2. The numbers of MCs were higher in fibrotic areas than in non-fibrotic areas in IPF (Table 2). MCs were seen especially in the areas of dense fibrosis and honeycombing while fibroblast foci contained less MCs. On average, the MC densities were 400 MCs/mm^2^ (SD 140) in the area of dense fibrosis, 530 MCs/mm^2^ (SD 110) in the honeycombing area, and 160 MCs/mm^2^ (SD 40) in fibroblast foci. Figure 1 presents the images of MCs in specimens of a patient with IPF, a control subject and a mouse isotype control. There were no significant interobserver differences in detected MC counts (intraclass correlation coefficients 0.998 between J.S. and S.L. and 0.999 between J.S. and M.K.).

**Table 2:** Mast cell densities in different lung tissue specimens

Specimen type	Mast cells per square millimeter, mean ± SD
Surgical lung biopsy	
UIP (*N* = 44)	321 ± 104
UIP with DAD (*N* = 3)	267 ± 149
Preserved alveolar areas in subjects with UIP (*N* = 32)	71 ± 50
Autopsy lung tissue specimen	
UIP with DAD (*N* = 7)	124 ± 102
Normal control (*N* = 12)	54 ± 17

*DAD* diffuse alveolar damage, *SD* standard deviation, *UIP* usual interstitial pneumonia

**Fig. 2:**
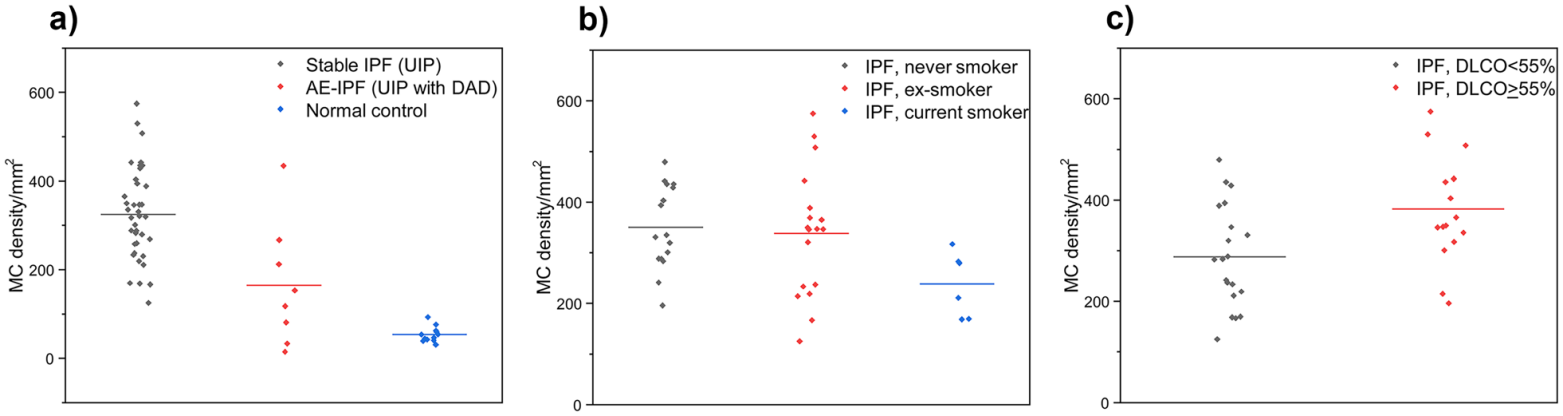
**a** Mast cell density was higher in fibrotic areas of stable idiopathic pulmonary fibrosis (IPF) patients compared with acute exacerbation of IPF (AE-IPF) patients or normal controls. **b** Mast cell density was higher in never or ex-smokers than in current smokers. **c** High mast cell density was associated with a high lung diffusion capacity for carbon monoxide (DLCO).

The MC densities in the fibrotic areas of lung tissue correlated with several clinical parameters, as shown in Table 3 and Fig. 2. Men had a higher MC density than women (*p* = 0.041) and current smokers had a lower MC density than ex- or non-smokers (*p* = 0.023). There was no association with forced vital capacity (FVC) and MC density, but in contrast, a high diffusion capacity for carbon monoxide (DLCO) was linked with a high MC density (*p* = 0.010). Patients with IPF had a higher MC density in their fibrotic lung tissue areas when compared with normal lung tissue derived from controls (*p* < 0.001), while the difference between non-fibrotic areas in IPF and controls was not statistically significant. We found no correlation of a future episode of AE-IPF with MC density in lung tissue biopsies taken in the stable phase of the disease.

**Table 3:** Mast cell parameters in IPF patients and normal controls

Characteristic (*N*/*N*)	Histology of the specimens included in analysis	Mast cells, mean ± SD	*P* value
Male/female (31/13)	UIP	342 ± 109/273 ± 72	0.041
Smoker/never-smoker (6/16)	UIP	238 ± 64/350 ± 83	0.007
Ever smoker/never-smoker (24/16)	UIP	313 ± 119/350 ± 83	0.287
Smoker/ex- and never-smokers (6/34)	UIP	238 ± 64/344 ± 105	0.023
FVC ≥ 75%/FVC < 75% (18/19)	UIP	304 ± 116/340 ± 97	0.302
DLCO ≥ 55%/DLCO < 55% (16/20)	UIP	382 ± 105/288 ± 101	0.010
IPF/controls (44/12)	UIP (fibrotic areas) and alveolar areas of normal controls	321 ± 104/54 ± 17	< 0.001
IPF patients with a future episode of AE-IPF/IPF patients not experiencing AE-IPF (18/26)	UIP	317 ± 113/325 ± 99	0.805
IPF/controls (32/12)	UIP (preserved alveolar areas) and alveolar areas of normal controls	71 ± 50/53 ± 17	0.261
IPF/AE-IPF (39/8)	UIP and UIP with DAD^a^	324 ± 103/165 ± 139	< 0.001

Data is presented as MC numbers per square millimeter ± SD. The MC densities of IPF patients recorded are related to the fibrotic areas of the lung tissue specimens, if not otherwise specified. *P* value was calculated with independent-samples *t*-test

*AE-IPF* acute exacerbation of idiopathic pulmonary fibrosis, *DAD* diffuse alveolar damage, *IPF* idiopathic pulmonary fibrosis, *UIP* usual interstitial pneumonia

^a^In two patients having undergone both biopsy and autopsy during the same episode of AE-IPF, the MC densities of biopsies were included in the analysis

### Mast Cell Density Was Low in Subjects with Diffuse Alveolar Damage

Patients with AE-IPF (i.e., UIP with DAD in lung tissue specimen) had a lower MC density than patients without AE at the time of biopsy (i.e., UIP without DAD) (*p* < 0.001) (Table 3, Fig. 2). We were able to demonstrate a significant decrease in MC density in five study subjects for whom we had lung tissue material obtained both at the time of diagnosis and later during an episode of AE-IPF (*p* = 0.010, Table 4).

**Table 4:** Patients in whom two separate lung tissue specimens had been obtained (both surgical lung biopsy and autopsy samples) in the stable phase and after an acute exacerbation of idiopathic pulmonary fibrosis

Patient	Age at biopsy (years)	Gender	FVC at biopsy (%)	Follow-up time from biopsy (months)	MCs/mm^2^ (biopsy, UIP without DAD)	MCs/mm^2^ (autopsy, UIP with DAD)
1	76	Female	60	4.3	196	81
2	49	Male	62	15	242	118
3	66	Male	44	34	480	268
4	49	Female	79	38	215	34
5	52	Male	No data	25	369	15
Total	58 ± 12		61 ± 14	25 (9.7 − 36)	300 ± 121^a^	103 ± 100^a^

Data is presented as mean ± standard deviation or median (interquartile range). Reported MC densities are determined from the fibrotic areas of the lung tissue specimens. *P* value was calculated by using paired sample *t*-test

*AE-IPF* acute exacerbation of idiopathic pulmonary fibrosis, *FVC* forced vital capacity, *MC* mast cell

^a^*P* value = 0.010 (between mast cell numbers in lung tissue specimens with or without DAD)

### Mast Cell Density, Survival, and Occurrence of AE-IPF

The correlation between the survival time and MC density was not statistically significant, although there was a slight trend towards a shorter survival and low MC density (319 MCs or less per square millimeter) (RR 1.59 95%CI 0.81 − 3.10, *p* = 0.175). MC densities in tissue specimens obtained at diagnosis in the stable phase of the disease, without clinical or histological evidence of AE-IPF at the time of biopsy date, were not correlated with the time to the first future episode of AE-IPF (RR 1.08 95% CI 0.43 − 2.74, *p* = 0.870).

## DISCUSSION

We studied the MC densities of lung tissue specimens including both fibrotic and non-fibrotic areas of 47 IPF patients and observed that the number of MCs was significantly higher in fibrotic areas as compared to non-fibrotic areas of IPF. We detected correlations of MC densities in fibrotic areas with several clinical parameters. Female gender, smoking, and low DLCO were associated with a low MC density. We also observed that the MC density was lower in lung tissue specimens obtained during an AE-IPF in comparison with lung tissue samples obtained at the time of diagnosis with no evidence of an AE. However, the baseline MC density did not correlate with either survival time or occurrence of an AE-IPF in the future.

In several previous studies, the number of MCs has been higher in patients with IPF than in control subjects, thus supporting our findings [6, 7, 9–12, 14, 15]. We were also able to demonstrate a higher MC density in the areas of dense fibrosis and honeycombing compared with areas of fibroblast foci, a finding supported by a previous study [6]. We detected a correlation between a high DLCO and male gender with a high MC density, and smoking with a low MC density, findings which have not been reported previously.

Male gender is a well-known risk factor for IPF [18]. Moreover, male gender has been related to shorter survival in IPF patients in some [19, 20], but not in all studies [21]. MC density was higher in male than in female patients in the present study, indicating that differences in MC numbers and functions might be partially responsible for the gender differences known to be associated with the risk and prognosis of IPF.

Smoking has also been identified as a risk factor for IPF [18], which was also evident in our study in the high proportion of ever smokers among IPF patients. Moreover, smoking has been assessed to be a prognostic factor in IPF, since, paradoxically, non- or ex-smokers seem to have a poorer prognosis than current smokers, at least in unadjusted models [22−24]. An association of MC density with smoking status has not been detected in previous studies investigating IPF patients. In a previous investigation, an increased number of MCs in bronchial mucosa has been observed in asymptomatic smokers compared with healthy controls [25]. However, in our study, the subjects’ smoking seemed to decrease MC counts rather than increase them. Concerning the ability of MCs to alter their phenotype and functions [26], it can be speculated that smoking might have different effects on the number and functions of MCs in non-fibrotic lungs compared with fibrotic lungs.

Some investigators have described an association of low PFT results (FVC or forced vital capacity in one second (FEV1)) with a high MC density [6, 13, 15], findings which we were unable to confirm. In contrast, there is one previous report of a slower rate of decline in FVC in patients with a high MC density [14]. This finding is consistent with our results, according to which patients with better preserved DLCO had a higher MC density than those with low DLCO.

The discrepancies between the results mentioned above might be partly due to the differences in MC staining techniques: Andersson et al. found the association of a high MC density with low FEV1% predicted only with MCs double positive for both chymase and tryptase [13]. Shimbori et al. used immunofluorescence staining for tryptase and chymase, not immunohistochemical staining as has been the more common practice, and found correlations of high MC numbers with low FVC with several different MC stainings [15].

In addition to the differences in MC staining techniques, inconsistent results concerning MC densities and their correlations with clinical parameters might also be related to the methods used to calculate and choose the tissue areas for MC quantification. The determination of one MC density value for the whole area of tissue sample does not take into account the varying proportions of fibrosis and spared alveolar tissue in different tissue specimens, a phenomenon typically present in IPF where fibrotic areas of lung tissue alternate with less-affected parenchyma [2]. Consistent with our findings, previous studies suggested that the MC density was much higher in the fibrotic area of the lung when compared with the spared alveolar area [9, 10, 13, 16], which highlights the importance of conducting separate MC quantifications for different compartments of lung tissue.

We could demonstrate a significant decline in the MC density of five patients who had undergone a lung biopsy for diagnostic purposes in the stable phase of disease and in whom an autopsy had been performed after death caused by an AE-IPF. No similar findings have been reported earlier, because there are very few studies involving multiple lung tissue specimens obtained from the same individual in different stages of pulmonary fibrosis.

To our knowledge, there are no previously published studies investigating lung MC density in AE-IPF patients. Animal models have suggested MCs and their mediators have a role in the pathogenesis of acute respiratory distress syndrome (ARDS), a phenomenon in which DAD is present similarly to AE-IPF [27−30]. However, this proposal has not been supported by clinical studies. Liebler et al. studied lung MCs in patients with different stages of ARDS indicating that MC numbers were not elevated in the early phase of ARDS compared with normal controls [31]. Based on their results, Liebler et al. speculated that MCs may not initiate the process of DAD and this might concern AE-IPF patients as well, because we could not find any association between MC numbers with the occurrence of AE-IPF, and during AE-IPF, MC numbers had declined, not become elevated. We cannot make any direct comparisons between our results and those of Liebler et al., because the lung MC densities between healthy patients and stable IPF patients differ significantly from each other according to several earlier studies [6, 7, 9–12, 14, 15, 31].

MCs are known to be activated by several different mechanisms, e.g., immune receptors, bacteria and their products, viruses, cytokines and inflammatory mediators, endogenic peptides, and physical stimuli [32]. MCs are postulated to change their phenotype and participate in wound healing processes [26]. It can be speculated that MC numbers and phenotypes might be changed by different stimuli also in damaged, fibrotic lung tissue during the course of the disease, i.e., MCs might have variable effects on pulmonary fibrosis in different stages of the disease. The variability of MC phenotypes might partly explain the association of MCs with pathogenesis of IPF as shown in several previous studies but these cells may not be the primary cell type playing the most significant role in the pathogenesis of AE-IPF [7, 11, 13, 15–17].

In the study by Overed-Sayer et al., nintedanib, but not pirfenidone, inhibited fibroblast-mediated MC survival in vitro and this finding was confirmed in the rat bleomysin model [6]. In our study, the low MC density of the patients with AE-IPF was not caused by nintedanib, because none of the patients had used antifibrotic therapy preceding the AE. Six out of seven patients with DAD in their lung tissue specimens had received corticosteroid treatment preceding SLB or autopsy, which might have reduced the number of MCs in lung tissue during AE-IPF. Those patients that had undergone SLB for diagnostic purposes were treatment naive, which means that baseline MC densities could not have been affected by antifibrotic or anti-inflammatory drugs. It is known that corticosteroids, which exert MC inhibiting effects, are not beneficial in the treatment of stable IPF and furthermore, the evidence of their efficacy in the treatment of AE-IPF is also limited [1−2]. These facts are in line with our results, according to which a high MC density was associated with a high DLCO during stable IPF and low MC density with AE-IPF. It can be speculated that MCs might have some protective effects on fibrotic lung tissue and inhibition of MCs might not be beneficial for patients. We were not able to detect any association of baseline MC density with the occurrence of AE-IPF. However, this result might have been affected by the limited study population and confounding factors, such as the use of nintedanib or corticosteroids during the follow-up time.

We were not able to find a statistically significant association of low MC density with short survival time, although a trend in this direction could be observed. It should be noted that we correlated survival time with MC density determined from patients’ SLB samples, of which only 3 were obtained during AE-IPF with a relatively low MC density. It was not reasonable to correlate MC cell densities of autopsy samples with survival time due to the lack of follow-up time of these patients. One may speculate that if we had had SLB specimens obtained slightly before death from a larger number of IPF patients, then it is possible that a correlation of low MC density and short survival time might have been found.

Overall, we managed to gather a rather comprehensive lung biopsy material from IPF patients, namely lung tissue specimens from 47 patients with additional autopsy material from 7 patients; as far as we are aware, this is the largest histological material on IPF patients from which an MC quantification has been performed. In earlier studies, the study material has mainly included tissue specimens from fewer than 20 IPF patients [9, 10, 12, 13, 16], with the exception of 3 studies in which the study populations were slightly larger i.e. lung tissue material from 21, 29 and 24 patients [7, 11, 14]. Although autolysis may have had some detrimental impact on the quality of autopsy specimens in our material, it did seem that our MC tryptase antibody produced results that were in line with those of biopsy specimens.

## CONCLUSIONS

To conclude, the MC density had significantly declined in subjects with an AE-IPF indicating that these cells may play variable roles in different stages of the disease. Altogether, the role of inflammation, MCs and other immune cells should be studied further in order to gain a more profound understanding of the pathobiology of IPF as this may make it possible to identify new targets for medical treatment for this serious disease.

## Supplementary Information

Below is the link to the electronic supplementary material.

Supplementary file1 (PDF 163 KB)

## Data Availability

The datasets generated during and/or analysed during the current study are available from the corresponding author on reasonable request.
