# Supplementary material for: Decline in Mast Cell Density During Diffuse Alveolar Damage in Idiopathic Pulmonary Fibrosis
Source: Inflammation. 2021 Oct 22;45(2):768–79. doi: 10.1007/s10753-021-01582-0 (PMC8956519; doi:10.1007/s10753-021-01582-0)
Supplement: Supplementary file 1 — Supplementary file1 (PDF 163 KB) [file 10753_2021_1582_MOESM1_ESM.pdf]

Supplementary Information

Decline in mast cell density during diffuse alveolar damage in idiopathic pulmonary fibrosis

Johanna Salonen*[a, b] (johanna.salonen@oulu.fi), Mervi Kreus[a, b], Siri Lehtonen[b, c], Hannu Vähänikkilä[d], Minna Purokivi[e], Riitta Kaarteenaho[a, b]

[a] Respiratory Medicine, Research Unit of Internal Medicine, University of Oulu, Oulu, Finland

[b] Medical Research Center (MRC) Oulu, Oulu University Hospital, Oulu, Finland

[c] Department of Obstetrics and Gynecology, Oulu University Hospital and PEDEGO Research Unit, University of Oulu, Oulu Finland

[d] Northern Finland Birth Cohorts, Arctic Biobank, Infrastructure for Population Studies, Faculty of Medicine, University of Oulu, Oulu, Finland

[e] The Center of Medicine and Clinical Research, Division of Respiratory Medicine, Kuopio University Hospital, Kuopio, Finland.

*Corresponding Author

TABLE 1. Studies on mast cells in patients with idiopathic pulmonary fibrosis (IPF) compared with other interstitial lung diseases (ILD) or healthy controls.

Study	Material	Study subjects	Staining technique for MCs	Findings
Hunt et al 1992	Open lung biopsy (IPF)<br>Autopsy lung specimen (control)	15 idiopathic pulmonary fibrosis (IPF)<br>6 normal controls	MC tryptase	Higher number of MCs in IPF compared with controls (counted areas were directly adjacent to the lumen of bronchioles).<br>Mast cells were detected particularly in the areas of dense fibrosis.<br>Altered appearance of mast cells in IPF (irregular surfaces and degranulation).
Pesci et al 1993	Transbronchial biopsies	9 cryptogenic fibrosing alveolitis<br>16 sarcoidosis<br>15 farmer's lung disease<br>6 idiopathic BOOP<br>3 histiocytosis X<br>7 control subjects (non-diseased area from lung cancer patients)	Toluidine blue	MC counts were higher in fibrotic lung disorders compared with control group.<br>The number of mast cells was positively correlated with the degree of fibrosis
Inoue et al 1996	Thoracoscopic biopsies<br>Transbronchial biopsies<br>Autopsy specimens<br>BAL<br>Tissue from lung allograft donors<br>Cultured human MC and human liver adenocarcinoma cell lines	21 IPF<br>23 chronic beryllium disease (CBD)<br>8 beryllium sensitized subjects without ILD<br>10 sarcoidosis patients (Sar)<br>18 normal controls	MC tryptase	MCs were found throughout the interstitium in IPF<br>The number of MCs was higher in ILD patients compared with controls, and higher in IPF than in Sar or CBD<br>Anti-basic fibroblast growth factor (anti-bFGF) bound to MCs in the lung interstitium<br>MCs containing bFGF accumulate in the areas of extracellular matrix deposition
Inoue et al 2002	Open lung biopsies<br>Fibrotic lung tissue from ILD patients having undergone transplantation<br>Autopsy specimens	14 IPF<br>9 lymphangioleiomyomatosis (LAM)<br>10 controls	MC tryptase	Higher number of basic fibroblast growth factor (bFGF) containing MCs in IPF and LAM compared with controls.<br>Association of MCs with a high degree of fibrosis in IPF and LAM

Hirata et al 2007	Snap-frozen specimens: Video-assisted thoracoscopic biopsies from lungs<br>One autopsy lung tissue specimen	7 usual interstitial pneumonia (UIP)<br>4 other ILD<br>8 normal controls	Both MC chymase and MC tryptase	The number of MCs was higher in idiopathic interstitial pneumonias than in control specimens.<br>The chymase-positive mast cells of ILD patients' lung tissue were related to an increase in interleukin-4-positive cells and an accumulation of smooth muscle cells and myofibroblasts
Andersson et al 2011	Lung tissue specimens collected in the association with lung transplantation or from open lung biopsy	5 Cystic fibrosis<br>7 IPF<br>8 controls (patients having undergone lung surgery for suspected cancer)	MC tryptase and chymase	MC density was increased in fibrotic areas of alveolar parenchyma in IPF<br>Density and percentage of MCTC correlated positively with the degree of fibrosis<br>Increased density of MCTCs as well as MC expression of TGF-β correlated negatively with patient lung function
Cha et al 2012	Lung tissue	29 IPF<br>16 chronic hypersensitivity pneumonia (cHP)<br>9 systemic scleroderma-associated ILD (SSc-ILD)<br>10 normal controls	MC chymase	A higher density of MCs in IPF compared with cHP, SSc-ILD or control.<br>IPF patients with high MC density had a slower rate of decline in forced vital capacity (FVC) than other ILD patients or controls.
Veerappan et al 2013	Animal model: Bleomycin induced fibrosis in mice<br>Human fibroblasts extracted from surgical lung biopsy and<br>BAL material	IPF (BAL and biopsies)<br>Normal controls (BAL and biopsies, surgical waste material)	Toluidine blue or glycoprotein avidin	MC-deficient mice were protected from bleomycin induced fibrosis<br>Re-introduction of MCs to MC-deficient mice led to pulmonary fibrosis<br>Both human and rat lung fibroblasts expressed histamine H1 and angiotensin II AT1 receptors<br>Histamine and renin were fibrogenic in an *in vitro* model
Wygrecka et al 2013	Lung tissue specimens from resected lungs of transplanted IPF patients<br>Bronchoalveolar lavage fluid (BAL)<br>In vitro cell cultures of human lung fibroblasts (HLF) and MCs	IPF (40 BAL and 24 lung tissue)<br>Normal controls (20 BAL and 10 non-used donor lungs)	MC tryptase	Elevated number of MCs in IPF compared with control subjects.<br>The majority of MCs in IPF lungs were activated.<br>HLFs induced MC activation and proliferation in vitro.<br>IPF patients' HLFs exhibited a higher growth rate compared with controls<br>Tryptase stimulated HLF growth and promoted extracellular matrix production in vitro.

Shimbori et al 2019	Lung tissue<br>Animal model: TGF-β1 or bleomycin induced pulmonary fibrosis in rats or mice	IPF<br>Normal lung tissue from patients having undergone surgery for lung cancer	Toluidine blue<br>Immunofluorescence stainings for tryptase and chymase	MC number was increased in IPF<br>MC number correlated inversely with lung function (forced vital capacity, all stainings)<br>MCs accumulated in fibrotic lung (animal model)<br>MCs on fibrotic extracellular matrix (ECM) produced more TGF-β compared with normal ECM (animal model)<br>Mechanical stretch of lungs caused mast cell degranulation
Overed-Sayer et al 2020	Lung biopsy material<br>Cell cultures with cord blood-derived MCs and normal human lung fibroblasts<br>Rats with bleomycin induced ILD	MC quantification and phenotyping:<br>17 IPF<br>9 chronic HP (cHP)<br>9 non-specific interstitial pneumonia (NSIP)<br>20 normal lung tissue from patients with lung cancer<br>MC protease signature analysis:<br>10 IPF lung volume reduction patients<br>9 normal lung tissue samples	MC tryptase and chymase	MCs were elevated in IPF and cHP lung, but not in NSIP<br>MCs correlated negatively with baseline FVC<br>MCs correlated positively with fibroblast foci<br>MC signature was increased in IPF lung<br>MCs induced fibroblast proliferation and activation<br>MCs and fibroblasts displayed crosstalk in co-culture studies<br>Nintedanib inhibited MC survival and activation<br>Nintedanib reduced MC recruitment in rat bleomycin model
Bagher et al 2021	Cell cultures on plastic plates and decellularized human lung tissue (scaffolds)	Human lung fibroblasts from IPF patients and healthy controls	NA	MCs and tryptase increased fibroblast release of mediators<br>Epithelial migration was influenced by the release of mediators

BAL, bronchoalveolar lavage; bFGF, basic fibroblast growth factor; BOOP, bronchiolitis obliterance organizing pneumonia; CBD, chronic beryllium disease; ECM, extracellular matrix; FVC, forced vital capacity; HLF, human lung fibroblast; HP, hypersensitivity pneumonia; ILD, interstitial lung disease; IPF, idiopathic pulmonary fibrosis; LAM, lymphangioleiomyomatosis; MC, mast cell; MCTC, mast cells positive for both tryptase and chymase, NSIP, non-specific interstitial pneumonia; sar, sarcoidosis; Ssc-ILD, systemic scleroderma-associated ILD; TGFβ1, transforming growth factor β1; UIP, usual interstitial pneumonia

TABLE 2. Clinical characteristics of normal controls compared with IPF patients.

	IPF (N=47)	Control (N=12)	P-value
Age, years	62±7.7	71±6.0	<0.01
Male	34 (72.3)	2 (16.7)	<0.01
Smoking status at biopsy			<0.01
Never-smoker	16 (38.1)	11 (91.7)	
Ex-smoker	20 (47.6)	1 (8.3)	
Current smoker	6 (14.3)	0	
Pack-years of ever-smokers[a]	26±11	7	0.115
FVC%[b]	74±16	106±14	<0.01
FEV1%[c]	79±17	105±15	<0.01
FEV1/FVC[d]	86±6.0	78±3.4	<0.01
DLCO%[e]	55±13	81±5.8	<0.01

Data is presented as mean± standard deviation, median (interquartile range) and number of patients (%) when appropriate. [a] Data of 13 smokers or ex-smokers was missing, [b] Data of 9 IPF patients and 4 control patients was missing, [c] Data of 8 IPF patients and 4 control patients was missing, [d] Data of 9 IPF patients and 5 control patients was missing [e] Data of 10 IPF patients and 8 control patients was missing.
